# Quality assurance in mental health: A case study of the Life Esidimeni tragedy

**DOI:** 10.4102/sajpsychiatry.v24i0.1267

**Published:** 2018-09-20

**Authors:** Lesley J. Robertson, Malegapuru W. Makgoba

**Affiliations:** 1 Department of Psychiatry, Faculty of Health Sciences, University of the Witwatersrand, South Africa; 2 Office of the Health Ombud, South Africa

## Abstract

**Introduction:**

Mental health has been included in the Sustainable Development Goals and quality assurance is needed to ensure its achievement. To date, more than 140 people with severe mental illness have died in the Life Esidimeni tragedy. The tragedy highlights the ineffectiveness of quality assurance in South Africa.

The aim of this case study was to identify possible measures which may be used for future monitoring of mental health by analysing the quality deficits evident in the Life Esidimeni tragedy.

**Methods:**

A framework for the analysis was devised using South African legislation and policy as well as international literature on quality assurance in mental health. Categories of quality care for macro-, meso- and micro-levels of analysis were developed. The Health Ombud’s report into the ‘Circumstances surrounding the deaths of mentally ill patients: Gauteng Province’ was examined for phrases which reflected quality care. These were allocated to the different categories and clustered into common themes which could be reflected as ‘quality measures’.

**Results:**

Measures identified at macro-level included basic human rights, mortality, financial protection and stakeholder consultation. Most quality deficits evident in the report were at meso-level and included adherence to legislation, access to health care and equitable financial distribution. At micro-level, transparent communication, treatment of medical conditions, relapse and readmission for mental illness, and caregiver and family satisfaction were identified. There were no references to user-reported quality measures in the report.

**Conclusion:**

In the context of South African legislation and policy, quality deficits at each level of analysis were exposed by the Life Esidimeni tragedy. These could be translated into health indicators or survey requirements for future quality assurance.

